# Misreporting and econometric modelling of zeros in survey data on social bads: An application to cannabis consumption

**DOI:** 10.1002/hec.3553

**Published:** 2017-08-04

**Authors:** William Greene, Mark N. Harris, Preety Srivastava, Xueyan Zhao

**Affiliations:** ^1^ Stern School of Business New York University New York NY USA; ^2^ Curtin Business School Curtin University Perth WA Australia; ^3^ School of Economics, Finance and Marketing Royal Melbourne Institute of Technology Melbourne VIC Australia; ^4^ Department of Econometrics and Business Statistics Monash University Melbourne VIC Australia

**Keywords:** cannabis consumption, discrete data, misclassification, ordered outcomes, zero‐inflated responses

## Abstract

When modelling “social bads,” such as illegal drug consumption, researchers are often faced with a dependent variable characterised by a large number of zero observations. Building on the recent literature on hurdle and double‐hurdle models, we propose a double‐inflated modelling framework, where the zero observations are allowed to come from the following: nonparticipants; participant misreporters (who have larger loss functions associated with a truthful response); and infrequent consumers. Due to our empirical application, the model is derived for the case of an ordered discrete‐dependent variable. However, it is similarly possible to augment other such zero‐inflated models (e.g., zero‐inflated count models, and double‐hurdle models for continuous variables). The model is then applied to a consumer choice problem of cannabis consumption. We estimate that 17% of the reported zeros in the cannabis survey are from individuals who misreport their participation, 11% from infrequent users, and only 72% from true nonparticipants.

## 1 INTRODUCTION AND BACKGROUND

Recreational drug use is one of the major social problems that policymakers face across the world, being associated with crime, violence, and more importantly, adverse health consequences. To this end, there exists a vast literature concerned with the empirical modelling of a wide array of data on drug use to help inform policymakers. Research on drug use and its consequences has arisen from several disciplines. Drug users' behaviour has been studied extensively in psychology, sociology, and medical arenas. In the last few decades, economists have also shown a growing interest in the study of drug use and its consequences. They have brought unique and useful perspectives to the understanding of drug users' behaviour, the onset of drug use, and abuse prevention, all of which have made important contributions to the drug policy debate. Crucial to effective policy analysis is the scope and quality of the drug data.

The availability of individual/household‐level drug data has brought an improved understanding of consumer behaviour. The analysis of differential policy responses by demographic characteristics, such as age, gender, and ethnicity, has been very useful for the development of drug policies and other educational programmes. Such data are invariably collected using survey techniques. The accuracy of the information gathered is dependent on reliable and accurate responses by the respondents. It may be the case, however, that respondents have an incentive to misreport their drug consumption given that drugs, in particular those of an illicit nature, are associated with legal risks, as well as stigma, or social disapproval. Such misreporting may lead to information being misclassified in survey data, which can mask the incidence of such behaviours and lead to biased and inconsistent estimates in statistical analyses (Hausman, Abrevaya, & Scott‐Morton, 1998). With an increasing use of survey data for drug policy analysis, it is therefore crucial to explore the incidence and implications of misreporting.

Although survey misclassification or misreporting (in the case of drugs and in general) is known to be pervasive and to potentially bias statistical/econometric analyses, there is a limited body of research that have explicitly modelled such behaviours. The relevant studies on misclassified dependent variables include Hausman et al. (1998), Abrevaya and Hausman (1999), Lewbel (2000), and Dustmann and van Soest (2001). Hausman et al. (1998) use a parametric approach to estimate misclassification probabilities when the functional form of the distribution of the error term is known. They consider a binary choice model with two types of misclassification: the probability that the true 0 is recorded as a 1; and vice versa. Abrevaya and Hausman (1999) and Lewbel (2000), on the other hand, consider a semiparametric approach to estimate the misclassification probabilities, where the distribution of the error term is unknown, except that the latter allows the misclassification probabilities to be covariate‐dependent functions. Dustmann and van Soest (2001) and, more recently, Greene, Harris, and Hollingsworth (2015) extend the parametric model of Hausman et al. (1998) to ordered data.

More recently, researchers such as Mahajan (2006), Hu (2008) and Molinari (2008) have attempted to model misclassification in discrete‐dependent variables using a secondary measurement or an instrument to identify a nonlinear model. Their approach is based on the observation that in the presence of classification errors, the relation between the true variable and its misclassified representation is given by a linear system of simultaneous equations in which the coefficient matrix is the matrix of misclassification probabilities. The so‐called anchoring, focal point answers, and crude rounding in surveys have also been a subject of interest among researchers (Kleinjans & van Soest, 2014; Manski & Molinari, 2010; van Soest & Hurd, 2012). Using a random‐effect multinomial logit model, Kleinjans and van Soest (2014) explicitly account for such reporting behaviour including nonresponse where respondents decide not to report any value. Finally, anchoring vignettes have also been used to measure discrepancies in reporting behaviours, particularly in the case of self‐reported health and life satisfaction (Kristensen & Johansson, 2008; van Soest, Delaney, Harmon, Kapteyn, & Smith, 2011); however, vignettes are very rare in most mainstream data sets.

This paper makes an important contribution to this received literature. The main aim is to develop a latent‐class or partial observability‐type modelling approach to analyse the extent of misreporting in drug consumption information collected using a large national drug survey. Our particular interest lies in misreporting in the context of cannabis consumption. However, the technique will also be generally applicable to empirical health models, such as sexual health and mental health, that involve sensitive responses and where there is a potential for inaccurate measurement of the variable(s) of interest. Essentially, we assume that for a “sensitive” response variable (such as drug use), there is an associated loss‐function (either perceived or actual, social and/or legal) involved for the individual in terms of the responses he/she reports. Here, it is clear that the researcher must be aware of the potential for misreporting. For example, there will be a strong incentive for individuals to misreport (presumably under‐report) their true consumption levels for fears of legal (and/or moral) repercussions (see, e.g., Pudney, 2007). This typically gives rise to a preponderance of “zero” observations in the data set.

There is a suite of *hurdle* and *double‐hurdle* models that have been developed over the years to address the build‐up of “zero” observations where the response variable is either a continuous variable with a nonzero probability mass at (typically, but not exclusively) zero levels (Cragg, 1971; Jones, 1989; Smith, 2003), or a count variable (Greene, 1994; Heilbron, 1989; Lambert, 1992; Mullahy, 1986; 1997; Pohlmeier & Ulrich, 1995), or an ordered discrete variable (Harris & Zhao, 2007). For example, in the zero‐inflated ordered probit (*Z* *I* *O* *P*) model, Harris and Zhao (2007) consider ordered levels of tobacco consumption and argue that the reported zeros could arise from both nonparticipants and infrequent consumers. In the same spirit, following the standard double‐hurdle arguments, we suggest that the build‐up of “zero” observations may correspond to both nonparticipants and participant (but infrequent) consumers. However, for these “social bads” with associated reporting loss‐functions, we also suggest a third source, involving those participants who, potentially due to fear of repercussions, report zero‐consumption when this in fact is not so.

This concept can be applied to the range of models mentioned above that exhibit a preponderance of zeros such as the zero‐inflated Poisson, *Z* *I* *P*, and other double‐hurdle model(s). Here, in view of our application to illicit drug use recorded on an ordinal scale, we focus on a *Z* *I* *O* *P* model; although the techniques can be similarly applied to other statistical models. Explicitly, we propose a three‐tiered approach: the first equation determines the participation decision; the second *conditional on participation*, determines whether an individual misreports; and finally, the third, *for participants who report accurately*, an ordered probit model determines the levels of consumption, which also include zero consumption of infrequent users. We term this generalisation of the *Z* *I* *O* *P* model, the double *Z* *I* *O* *P*(*D* *Z* *I* *O* *P*) model. In research in areas of discrete random variables that are inherently ordered, misreporting has sometimes been addressed by allowing the model's inherent boundary parameters to vary by observed personal characteristics (Greene & Hensher, 2010). Here, in addition to the “fundamental” form of modelling misreporting, we can also allow for more general under‐ (or over‐) reporting, by allowing the boundary parameters to vary by observed characteristics.

## 2 THE ECONOMETRIC FRAMEWORK

### 2.1 A D Z I O P model

Our approach entails a fundamental form of modelling the misreporting, which is likely to be present in data that are perceived to embody a strong loss‐function (social and/or legal) for the individual. Following the *Z* *I* *O* *P* model of Harris and Zhao (2007), we start by defining a discrete random variable *y* that is observable and assumes the discrete ordered values of 0,1,…,*J*. A standard *O* *P* approach would map a single latent variable to the observed outcome *y* via so‐called boundary parameters, with the latent variable being related to a set of covariates (Greene & Hensher, 2010). However, the *Z* *I* *O* *P* model involves two latent equations: a probit selection equation and an *O* *P* equation. As with the more traditional double‐hurdle models (Jones, 1989), individuals here have to overcome two hurdles before one observes nonzero consumption: whether to participate, and then, conditional on participation, the amount to consume, *which also includes zero consumption*.

However, it is our contention here that, especially regarding the consumption of “social bads” (e.g., licit and, in particular, illicit drugs), participants may intentionally misreport their true consumption patterns. In particular, we hypothesise that a (probably significantly large) proportion of participants may under‐report their true consumption levels by simply stating zero consumption. That is, we contend that, for example, if a user is concerned with legal ramifications of admitting drug use, he/she will typically prefer to misreport “none at all,” as compared to simply under‐reporting their true use. The alternative assumption would be akin to someone feeling more comfortable to admitting breaking the law “by just a little.”

Finally, participants who do not misreport, as with the *Z* *I* *O* *P* model, are free to select any of the *j*=0,…,*J* outcomes. In this way, observed zero‐consumption can arise from the following: (a) nonparticipants; (b) participants who misreport; or (c) participants who do not misreport, but who are infrequent consumers (e.g., who happen to not have used drugs in the past 12 months). Thus, as compared to say, a standard *O* *P* approach, the zero observations are “double‐inflated”: once by nonparticipants and then by misreporters. We suggest a three‐tiered sequencing of decision making. First, the individual makes a decision whether to participate or not; secondly, there is the decision to misreport or not; and finally, there is the decision on how much to consume.

Following Harris and Zhao (2007), we let *r* denote a binary variable indicating the split between Regime 0 (with *r*=0 for nonparticipants) and Regime 1 (with *r*=1 for participants). Although unobservable, *r* is related to a latent variable *r* ^∗^ via the mapping: *r*=1 for *r* ^∗^>0 and *r*=0 for *r* ^∗^⩽0. The variable *r* ^∗^ represents the propensity for participation. It is related to a set of explanatory variables $\left(x_{r}\right)$ with unknown weights ***β***_*r*_ and a standard normally distributed error term *ε*_*r*_ such that

(1) $$r^{*}=x_{r}^{\prime }\beta_{r}+\varepsilon_{r}.$$

A second latent variable *m* ^∗^ represents the propensity to misreport. Again, this is related to a second unobserved variable *m* such that *m*=1 for *m* ^∗^>0 and *m*=0 for *m* ^∗^⩽0, where *m*=0 represents a misreporter and *m*=1 a truthful reporter. Again, we can write this as a linear latent form as

(2) $$m^{*}=x_{m}^{\prime }\beta_{m}+\varepsilon_{m}.$$

Finally, consumption levels under Regime 1 are represented by a discrete variable $\tilde{y}$ $\left(\tilde{y}=0,1,\text{⋯},J\right)$ generated by an *O* *P* model via a third latent variable ${\tilde{y}}^{*}$ such that

(3) $${\tilde{y}}^{*}=x_{y}^{\prime }\beta_{y}+\varepsilon_{y},$$

with the standard mapping of

(4) $$\tilde{y}=\left\{\begin{array}{cc} 0 & \;\text{if}\;{\tilde{y}}^{*}⩽0,\\ j & \;\text{if}\;\mu_{j-1}<{\tilde{y}}^{*}⩽\mu_{j},(j=1,\text{⋯},J-1),\\ J & \;\text{if}\;\mu_{J-1}⩽{\tilde{y}}^{*}, \end{array}\right.$$

where ***μ*** is a vector of boundary parameters to be estimated (the extreme values, *μ*_0_ and *μ*_*J*_, are normalised at 0 and +∞, respectively). Of course, as with the *Z* *I* *O* *P* model, $\tilde{y}$ is not directly observed. Nor is either *r* or *m*. Here, the observability criterion for observed *y* is

(5) $$y=r\times m\times \tilde{y}.$$

An observed *y*=0 outcome can arise from three distinct sources: *r*=0 (the individual is a nonparticipant); *r*=1 (the individual is a participant) and jointly that *m*=0(the individual is a misreporter); and finally, that jointly *r*=1, *m*=1, and $\tilde{y}=0$ (the individual is a zero consumption, accurate‐reporting participant). In the same way, to observe a positive *y*, we require jointly that the individual is a participant (*r*=1) and an accurate reporter (*m*=1) and that ${\tilde{y}}^{*}>0$. This setup is one of partial observability in line with models proposed by Poirier (1980) and Meng and Schmidt (1985).

For the time‐being, assume that the stochastic terms ***ε*** $\left(=\varepsilon_{r},\varepsilon_{m},\varepsilon_{y}\right)$ are independent and follow standard Gaussian distributions. The full probability for *y*=0 is given by

(6) $$\begin{array}{ccc} \mathrm{Pr}(y=0\left|x\right.) & =\mathrm{Pr}\left(r=0|x\right) & \\ & \;+\mathrm{Pr}(r=1|x)\mathrm{Pr}(m=0|x)\\ & \;+\mathrm{Pr}(r=1|x)\mathrm{Pr}(m=1|x)\mathrm{Pr}\left(\tilde{y}=0|x\right) \end{array}$$

and for the remaining outcomes

(7) $$\mathrm{Pr}\left(\;y=j|x\right)=\mathrm{Pr}\left(r=1|x\right)\mathrm{Pr}\left(m=1|x\right)\mathrm{Pr}\left(\tilde{y}=j|x\right)\;(\;j=1,\text{⋯},J).$$

By independence, these joint probabilities are simply products of the marginals such that, under the usual assumption of normality, they are given respectively by

$$\begin{array}{ccc} \mathrm{Pr}\left(\;y=0|\left|\;x\right.\right) & =\left[1-\Phi \left(x_{r}^{\prime }\beta_{r}\right)\right] & \\ & \;+\Phi \left(x_{r}^{\prime }\beta_{r}\right)\left[1-\Phi \left(x_{m}^{\prime }\beta_{m}\right)\right]\\ & \;+\Phi \left(x_{r}^{\prime }\beta_{r}\right)\Phi \left(x_{m}^{\prime }\beta_{m}\right)\Phi \left(-x_{y}^{\prime }\beta_{y}\right) \end{array}$$

and

(8) $$\begin{array}{ccc} \mathrm{Pr}\left(\;y=j|x\right) & =\Phi \left(x_{r}^{\prime }\beta_{r}\right)\Phi \left(x_{m}^{\prime }\beta_{m}\right)\left[\Phi \left(\mu_{j}-x_{y}^{\prime }\beta_{y}\right)-\Phi \left(\mu_{j-1}-x_{y}^{\prime }\beta_{y}\right)\right]\;(j=1,\text{⋯},J-1), & \\ \mathrm{Pr}\left(\;y=J|x\right) & =\Phi \left(x_{r}^{\prime }\beta_{r}\right)\Phi \left(x_{m}^{\prime }\beta_{m}\right)\left[1-\Phi \left(\mu_{J-1}-x_{y}^{\prime }\beta_{y}\right)\right]. \end{array}$$

The probability of a zero observation has been “double‐inflated” as it is a combination of the probability of “zero consumption” from the *O* *P* process and the probability of “nonparticipation” from the split probit model plus that from misreporting. Note that as per the *Z* *I* *O* *P* model, there may or may not be overlaps with the variables in the partitions in **x**_*r*_,**x**_*m*_, and **x**_*y*_, although undoubtedly identification will be aided by such.

Given the assumed form for the probabilities and an *i*.*i*.*d*. sample of size *N* from the population on (*y*_*i*_,**x**_*i*_), *i*=1,…,*N*, the parameters of the full model $\theta ={\left(\beta^{{}^{\prime }},\mu^{\prime }\right)}^{\prime }$(where $\beta =\beta_{r}\cup \beta_{m}\cup \beta_{y}$) can be consistently and efficiently estimated using maximum likelihood techniques. The log‐likelihood function is

(9) $$\ell \left(\theta \right)=\overset{N}{\underset{i=1}{\sum }}\overset{J}{\underset{j=0}{\sum }}h_{ij}\ln\left[\mathrm{Pr}\left(y_{i}=j|\left|\;x,\theta \right.\right)\right],$$

where the indicator function *h*_*ij*_ is

(10) $$h_{ij}=\left\{\begin{array}{c} 1\;\text{if individual}\;i\;\text{chooses outcome}\;j\\ 0\;\text{otherwise.} \end{array}\right.\;(i=1,\text{⋯},N;\;j=0,1,\text{⋯},J).$$

Maximization was performed using the Broyden–Fletcher–Goldfarb–Shanno algorithm (Greene, 2008). Robust standard errors were computed based on the common “sandwich” estimator. Standard errors of secondary estimated quantities, such as partial effects and summary probabilities, were estimated using the *Delta* method. 1Estimation computer code is available from the authors on request.Clearly, to apply a similar set‐up to count or continuous dependent variables, one could simply replace the *O* *P* densities above with the appropriate ones for the data at hand. 2Note the above model shows some similarities to that considered by Kasteridisa, Munkinb, and Yen (2010), although their focus was on smoking cessation decisions.


### 2.2 Generalising the model to correlated disturbances (D Z I O P C)

As described above, the observed realisation of the random variable *y* can be viewed as the result of three separate latent processes with uncorrelated error terms. However, these three outcomes correspond to the same individual, so it is likely that the vector of stochastic terms ***ε***_*i*_ will be related across equations. We now extend the model to have $\left(\varepsilon_{r},\varepsilon_{m},\varepsilon_{y}\right)$ follow a multivariate normal distribution with covariance matrix Ω_3_, whilst maintaining usual probit normalisations and unit variances. The full observability criteria are thus

(11) $$y=rm\tilde{y}=\left\{\begin{array}{cc} 0 & \;\text{if}\;(r^{*}⩽0)\;\text{or}\left(r^{*}>0\;\text{and}\;m^{*}⩽0\right)\;\text{or}\;\left(r^{*}>0\;\text{and}\;m^{*}>0\;\text{and}\;{\tilde{y}}^{*}⩽0\right)\\ j & \;\text{if}\;\left(r^{*}>0\;\text{and}\;m^{*}>0\;\text{and}\;\mu_{j-1}<{\tilde{y}}^{*}⩽\mu_{j}\right)\;(j=1,\text{⋯},J-1),\\ J & \;\text{if}\;\left(r^{*}>0\;\text{and}\;m^{*}>0\;\text{and}\;\mu_{J-1}<{\tilde{y}}^{*}\right), \end{array}\right.$$

which translate into the following expressions for the probabilities

(12) $$\mathrm{Pr}(y)=\left\{\begin{array}{c} \mathrm{Pr}\left(y=0|x\right)=\left[1-\Phi \left(x_{r}^{\prime }\beta_{r}\right)\right]+\Phi_{2}\left(x_{r}^{\prime }\beta_{r},-x_{m}^{\prime }\beta_{m};\Omega_{2}\right)+\Phi_{3}\left(x_{r}^{\prime }\beta_{r},x_{m}^{\prime }\beta_{m},-x_{y}^{\prime }\beta_{y};\Omega_{3}\right)\\ \mathrm{Pr}\left(y=j|x\right)=\Phi_{3}\left(x_{r}^{\prime }\beta_{r},x_{m}^{\prime }\beta_{m},\mu_{j}-x_{y}^{\prime }\beta_{y};\Omega_{3}\right)-\Phi_{3}\left(x_{r}^{\prime }\beta_{r},x_{m}^{\prime }\beta_{m},\mu_{j-1}-x_{y}^{\prime }\beta_{y};\Omega_{3}\right)\\ \mathrm{Pr}\left(y=J|x\right)=\Phi_{3}\left(x_{r}^{\prime }\beta_{r},x_{m}^{\prime }\beta_{m},x_{y}^{\prime }\beta_{y}-\mu_{J-1};\Omega_{3}\right), \end{array}\right.$$

where Φ_3_(.) and Φ_2_(.), respectively, denote the *c.d.f.* of the standardised trivariate and bivariate normal distribution and where Ω_2_ is the relevant submatrix of the full Ω_3_ matrix.

Maximum likelihood estimation would again involve maximization of Equation (9) replacing the probabilities of (8) with those of (12) and redefining ***θ*** as $\theta ={\left(\beta^{\prime },\mu^{\prime },\Omega_{3}\right)}^{\prime }$. 3We evaluate these trivariate probabilities using the GHK simulator.A test of Ω_3_=*I*_3_ is a joint test for independence of the three error terms and thus a test of the more general model given by Equation (12) against the null of a simpler nested model of Equation (8).

## 3 AN APPLICATION TO CANNABIS CONSUMPTION

Cannabis use imposes a high social and economic cost on society and has been a major concern to policy‐makers worldwide. It is the most commonly used drug after tobacco and alcohol, particularly in the younger population. A large amount of public funds have flowed into promotional campaigns and rehabilitation programmes in many countries across the world in order to treat and prevent cannabis‐related harm. This has resulted in a growing importance of research in order to develop sound policies and strategies. The quality of the evidence from these scientific investigations is an important concern, however. Because cannabis possession and market transactions constitute illegal activities in most jurisdictions, there is a strong incentive for cannabis users to conceal their behaviour, for fear of punishment. The concealment of cannabis use can also result from embarrassment or social disapproval (Swadi, 1990). Such misreporting can have a significant impact on the quality of research findings. A major focus of this paper therefore is to examine the profile of those people who misreport their cannabis consumption.

### 3.1 The data

The data we use for the model are drawn from the Australian National Drug Strategy Household Survey, which is a nationally representative survey of the noninstitutionalised Australian civilian population aged over 14 providing information on drug use patterns, attitudes, and behaviour (NDSHS, 2010). A multi‐stage, stratified area sample design ensured a random sample of households in each geographical stratum. As mentioned above, there has been some discussion in the existing literature regarding the potential for misreporting to be influenced by how the survey is conducted. The earlier waves of the National Drug Strategy Household Survey used face‐to‐face and drop‐and‐collect methods to collect data. The computer‐assisted telephone interview (CATI) method of data collection was introduced in the 2001 survey. In that particular survey, all three methods were employed to collect data. The 2004 and 2007 surveys, on the other hand, were administered using only the drop‐and‐collect and CATI methods, whereas the more recent surveys were conducted only using the CATI method.

We restrict our study to the 2001, 2004, and 2007 surveys for the following reasons. The older surveys included inconsistent questions with regard to the key variable of interest, whereas the more recent surveys were conducted using only the CATI method. As discussed later in the paper, variation in the method of collection is key to identifying the misreporting equation. Definitions of all variables used in the study are given in the Appendix. A sample of 50,153 individuals is thus available for estimation. This data set has been used in several previous studies (see, e.g., Cameron & Williams, 2001; Harris & Zhao, 2007; Williams, 2004; Zhao & Harris, 2004).

In this data set, neither the monetary expenditures on nor the physical quantities of cannabis consumed are reported. The information on individuals' consumption of cannabis is given via a discrete variable measuring the participation and intensity of consumption in the last 12 months. In particular, the information in the data concerning an individual's consumption of cannabis is collected through the question “*Have you used cannabis/marijuana in the last 12 months*” and “*In the last 12 months, how often did you use cannabis/marijuana?”,* where the responses to the frequency of use take the form of one of the following choices: *not at all* (*y*=0); *using cannabis once or twice a year*(*y*=1); *using cannabis monthly or every few months* (*y*=2); and *using cannabis everyday or once a week* (*y*=3).

In terms of explanatory variables, we have three blocks: **x**_*r*_, to determine participation; **x**_*m*_, for misreporting; and **x**_*y*_, to determine consumption levels. Although many of the variables overlap (as we have no *a priori* information as to where they should appear in the model and where not), to facilitate identification, we apply some natural exclusion restrictions. The common variables in the three equations include a wide range of personal and demographic characteristics, namely, gender; marital status; individual's (standardised) age; a dummy variable for whether there are preschool children in the household; whether the individual comes from a single parent household; a dummy variable for whether the individual resides in a capital city; and a dummy variable for whether the individual is of Aboriginal or Torres Strait Islander origin.

We also control for educational attainment, distinguishing between four categories of highest educational attainment: a tertiary degree; a nontertiary diploma or trade certificate; year 12 education; and less than year 12 education, which is the omitted category. Illicit drugs are just market commodities, and users are just market participants. In terms of the individual's economic situation, we control for the household annual income before tax measured in Australian dollars using eight income bands as described in the Appendix, with the highest band being the omitted category. Although income may act as a social class proxy in the participation and misreporting decisions, the amount of consumption is likely to be directly related to the level of income as it is with any normal good. We also use individual's main labour market status to control for their economic situation, that is, employed, studying, unemployed, and other activities such as retired, on a pension or performing home duties, which form the omitted category.

The criminal justice environment is an important determinant of drug participation and consumption. At the same time, it also increases the incentive to misreport. For instance, the fear of punishment may be heightened if users perceive that supplying accurate information could lead to legal repercussions. Australia has long‐standing laws with regard to cannabis decriminalisation. South Australia was the first jurisdiction to implement an expiation system for minor cannabis offences in 1987. Under this scheme, simple cannabis offences such as possessing or cultivating small amounts for personal use are subject to minor penalties, although the sanctions for commercial dealings are rather significant. Similar expiation systems have since been introduced in other Australian states and territories, and yet, others have been gradually easing their laws against cannabis consumption in recent years. We therefore include in all three equations a variable to represent the decriminalisation status of cannabis use across the various Australian states and territories. We also control for any migration effect by using an indicator for whether the individual has migrated to Australia in the last 10 years. Any time trend in participation and levels of consumption is addressed using time indicators for the surveys.

Although the inherent nonlinearity in our model can help achieve identification, we impose exclusion restrictions to ensure that the model is identified on data. 4For identification by functional form and related issues, see, for example, Li, Poskitt, and Zhao (2016).We therefore include additional explanatory variables in the participation equation, which we believe do not directly influence misreporting behaviour. In particular, drug culture or peer drug use has been identified as an important risk factor for drug participation (see, e.g., Delaney, Harmon, & Wall, 2008; Kenkel, Reed III, & Wang, 2002; Pudney, 2004). We therefore include the variable “peer” in **x**_*r*_ that represents the proportion of the individual's friends and acquaintances that use cannabis. This variable is excluded from the misreporting equation. Given evidence on the gateway effect of alcohol to harder drugs such as cannabis (Pacula, 1998) and the association between body piercing and tattoo procedures with risk‐taking behaviours (see, e.g., Deschesnes, Finès, & Demers, 2006; Heywood et al., 2012), we also include in **x**_*r*_ dummy variables indicating whether an individual started drinking alcohol at a young age (i.e., below the legal age of 18 years), and whether the individual has ever undergone a body piercing procedure or a tattoo procedure. These risk indicators are not expected to influence individuals' misreporting behaviour. We also include year dummies in **x**_*r*_ and **x**_*y*_  to represent any trend changes over time. Finally, an individual's attitude towards drug laws is very likely to influence his or her consumption. We thus include a dummy variable in **x**_*r*_ and **x**_*y*_, which takes the value 1 if the individual believes that a small quantity of cannabis for personal use should be a criminal offence, and 0 otherwise. These regressors are also excluded from **x**_*m*_.

We also use some exclusion restrictions to help identify the misreporting equation by including some variables exclusively in **x**_*m*_. Following the previous literature, these mostly relate to the conditions under which the survey was administered, and therefore may potentially influence the extent to which individuals misreport, but not their participation or consumption levels. Specifically, we control (using indicators) for the following: if anyone else was present when the respondent was completing the survey questionnaire (“present”); if anyone helped the respondent complete the survey questionnaire (“help”); and the survey format (“survtype”; which takes a value 0 if the drop‐and‐collect method was used and takes a value 1 if the CATI or face‐to‐face method was used). These variables conform with the factors that have been associated with misreporting or misclassification in prior studies (see, e.g., Berg & Lien, 2006; Hoyt & Chaloupka, 1994; Kraus & Augustin, 2001; Lu, Taylor, & Riley, 2001; Mensch & Kandel, 1988; O'Muircheartaigh & Campanelli, 1998), although none of these studies have modelled misreporting explicitly. We also include as an instrument a variable indicating a general lack of trust in the survey, which we proxy by the percentage of compulsory questions left unanswered in the survey. Note that nonresponse rates in general with regard to the response variable were very low (under 2%), such that this is not likely to adversely affect our approach and/or findings.

Finally, in terms of consumption levels, a standard consumer demand framework applies with special characteristics for addictive goods (see, e.g., Becker & Murphy, 1988). We thus include standard demand‐schedule own and cross‐drug prices in **x**_*y*_. Other than cannabis price, we control for the price of a range of related drugs such as amphetamines, cocaine, heroin, alcohol, and tobacco, in light of the evidence that certain drugs act as either complements or substitutes to cannabis (see, for example, Cameron & Williams, 2001; Ramful & Zhao, 2009; Zhao & Harris, 2004).

Price series for cannabis, cocaine, amphetamines, and heroin are obtained from the Illicit Drug Reporting System (IDRS) (NDARC, 2009). They vary across Australian states and territories and by year. The IDRS collects such data predominantly from interviewing injecting drug users and key informants who have regular contact with illicit drug users but which may potentially exhibit coverage error (NDARC, 2009). In occasional cases where a price report is missing, it is constructed using information from the Australian Bureau of Criminal Intelligence, which was replaced by the Australian Crime Commission in recent years. The Australian Bureau of Criminal Intelligence/Australian Crime Commission is an alternative source for drug prices, which collects information on drugs through covert police units and police informants (ACC, 2010).

The advantage of using price data from the IDRS is that they are provided with unified measures and fewer missing observations. To be specific, the price of cannabis is measured in dollars per ounce, and the respective price(s) of amphetamines, cocaine and heroin are measured in dollars per gram. The data on alcohol and tobacco prices are obtained in the form of indices from the Australian Bureau of Statistics (ABS, 2010). All price and income series are deflated using the all‐items Consumer Price Index (CPI) for individuals' respective states of residence. Clearly dependent upon the particular price series, there is a potential for measurement error here (especially with regard to the illegal drugs).

Table 1 presents some summary statistics on the observed cannabis consumption. On average, around 89% of individuals identify themselves as current nonusers. Given the way the survey questions are asked, these self‐identified nonusers or the *build‐up of zero observations* will include genuine nonusers, recent quitters, infrequent users who are not *currently* consuming cannabis, and potential users who might use when, say, the price falls. More importantly, these self‐identified nonusers may include misreporters who, out of embarrassment, social disapproval, and/or fear of repercussions, may prefer to identify themselves as nonusers, for example. Given (a) that users have incentives to misreport consumption and (b) that for users who report truthfully, the choices of consumption intensities are ordered, then this presents a good case for the *D* *Z* *I* *O* *P*(*C*) model(s) in order to identify the different types of zero observations and their potentially differing driving factors. Note that there is also the possibility of over‐reporting, particularly with regard to the intensity of consumption (possibly due to memory issues). However, there is evidence that over‐reporting is rarely a problem when analysing self‐reported drug use (see, e.g., Swadi, (1990), and references therein).

**Table 1:** Descriptive statistics

Variable	Mean	SD	Min	Max	# Obs.
Dependent variable					
*y*=0	0.888	‐	‐	‐	43472
*y*=1	0.036	‐	‐	‐	1740
*y*=2	0.034	‐	‐	‐	1680
*y*=3	0.042	‐	‐	‐	2078
Explanatory variables					
MALE	0.466	0.499	0	1	48970
STAGE	−0.016	0.916	−1.716	2.903	48970
STAGESQ	−0.045	0.917	−1.244	4.137	48970
MARRIED	0.625	0.484	0	1	48970
PRESCHOOL	0.132	0.338	0	1	48970
SINGPAR	0.067	0.251	0	1	48970
CAPITAL	0.644	0.479	0	1	48970
ATSI	0.012	0.110	0	1	48970
WORK	0.606	0.489	0	1	48970
STUDY	0.061	0.240	0	1	48970
UNEMP	0.023	0.149	0	1	48970
OTHER	0.310	0.463	0	1	48970
DEGREE	0.275	0.447	0	1	48970
YR12	0.126	0.332	0	1	48970
DIPLOMA	0.347	0.476	0	1	48970
LESSYR12	0.252	0.434	0	1	48970
HINC1	0.057	0.232	0	1	48970
HINC2	0.118	0.322	0	1	48970
HINC3	0.074	0.262	0	1	48970
HINC4	0.139	0.346	0	1	48970
HINC5	0.165	0.371	0	1	48970
HINC6	0.193	0.395	0	1	48970
HINC7	0.077	0.267	0	1	48970
HINC8	0.177	0.382	0	1	48970
DECRIM	0.256	0.437	0	1	48970
MIGR10	0.047	0.211	0	1	48970
YNGDRINK	0.597	0.490	0	1	48970
YR04	0.355	0.479	0	1	48970
YR07	0.320	0.466	0	1	48970
YR10	0.325	0.468	0	1	48970
TATTOO	0.105	0.307	0	1	48970
BODYPIER	0.077	0.267	0	1	48970
PEER	0.042	0.202	0	1	48970
YGPMAR	0.155	0.890	0	5.474	48970
YGPTOB	0.165	0.942	0	5.646	48970
CRIMSUP	0.290	0.454	0	1	48970
PRESENT	0.298	0.458	0	1	48970
HELP	0.231	0.421	0	1	48970
SURVTYPE	0.179	0.383	0	1	48970
TRUST	0.035	0.051	0	0.610	48970
LRPMAR	5.238	0.155	4.809	5.474	48970
LRPCOC	5.182	0.224	4.818	5.824	48970
LRPHER	5.524	0.335	4.831	6.348	48970
LRPSPD	4.660	0.476	3.514	5.346	48970
LRPTOB	5.560	0.051	5.450	5.646	48970
LRPALC	4.712	0.036	4.630	4.766	48970

### 3.2 The results

Table 2 reports the estimated coefficients of the *D* *Z* *I* *O* *P* *C* model. In particular, we report three sets of results corresponding to the three equations: participation, truthful reporting, and levels of consumption. Note that out of three correlation coefficients, only *ρ*_12_(i.e., the correlation between the participation and misreporting equations) is strongly statistically significant (although the joint Likelihood Ratio test only marginally fails at 10%). 5Nonetheless, we present the correlated DZIOP results as they are essentially the same as the uncorrelated ones.


**Table 2:** DZIOPC coefficient estimates

				Truthful			Levels of	
	Participation			reporting			consumption	
CONSTANT	−1.556	(0.115)**		1.839	(0.270)**		−10.460	(6.264)*
STAGE	−0.749	(0.037)**		0.660	(0.130)**		1.609	(0.167)**
STAGESQ							−1.897	(0.192)**
MALE	0.130	(0.043)**		0.283	(0.094)**		0.431	(0.042)**
MARRIED	−0.380	(0.045)**		0.011	(0.112)		−0.030	(0.057)
PRESCHOOL	−0.093	(0.059)		−0.069	(0.130)		−0.209	(0.057)**
SINGPAR	0.082	(0.067)		−0.218	(0.126)*		−0.101	(0.064)
CAPITAL	−0.089	(0.041)**		0.319	(0.089)**		0.031	(0.046)
ATSI	0.359	(0.137)**		−0.525	(0.171)**		0.283	(0.132)**
WORK	0.050	(0.057)		0.127	(0.133)		−0.215	(0.065)**
STUDY	−0.069	(0.095)		0.241	(0.179)		−0.317	(0.090)**
UNEMP	0.142	(0.088)		0.380	(0.212)*		0.163	(0.099)*
DEGREE	0.312	(0.059)**		−0.454	(0.139)**		−0.468	(0.070)**
DIPLOMA	0.165	(0.046)**		−0.201	(0.113)*		−0.160	(0.054)**
YR12	0.137	(0.059)**		−0.125	(0.134)		−0.152	(0.060)**
HINC1	0.045	(0.164)		−0.364	(0.316)		0.390	(0.173)**
HINC2	0.147	(0.110)		0.277	(0.327)		0.230	(0.130)*
HINC3	0.036	(0.080)		0.060	(0.176)		0.415	(0.080)**
HINC4	0.084	(0.079)		−0.044	(0.173)		0.348	(0.083)**
HINC5	−0.016	(0.063)		0.036	(0.149)		0.380	(0.062)**
HINC6	−0.003	(0.057)		−0.083	(0.136)		0.242	(0.057)**
HINC7	−0.039	(0.053)		0.103	(0.140)		0.130	(0.054)**
DECRIM	0.152	(0.042)**		−0.313	(0.091)**		0.046	(0.055)
MIGR10	−0.212	(0.091)**		0.362	(0.314)		0.050	(0.094)
YNGDRINK	0.590	(0.039)**					0.221	(0.093)**
YR04	−0.012	(0.031)					0.057	(0.067)
YR07	−0.101	(0.033)**					0.016	(0.103)
TATTOO	0.305	(0.035)**					0.128	(0.050)**
PIERCING	0.437	(0.046)**					0.144	(0.059)**
PEER	1.560	(0.071)**					1.001	(0.124)**
CRIMSUP	−1.049	(0.082)**					−0.985	(0.150)**
PRESENT				−0.220	(0.074)**			
HELP				−0.240	(0.091)**			
SURVTYPE				−0.264	(0.108)**			
TRUST				−2.179	(0.630)**			
LRPMAR							0.420	(0.136)**
LRPCOC							−0.029	(0.099)
LRPHER							0.230	(0.101)**
LRPSPD							−0.098	(0.059)*
LRPTOB							0.761	(0.664)
LRPALC							0.756	(0.604)
*μ*_1_							0.819	(0.074)**
*μ*_2_							1.568	(0.097)**
Estimated correlation coefficients:^+^								
*ρ*_*r*,*m*_	−0.591 (0.135)**					
$\rho_{r,\tilde{y}}$	0.038 (0.158)					
$\rho_{m,\tilde{y}}$	0.280 (0.177)					

*Note*. ^+^ *ρ*_*a*,*b*_ with $a,b\in \left(r,m,\tilde{y}\right)$ correspond to the correlation coefficients across the participation (*r*), miseporting (*m*), and level of consumption $\left(\tilde{y}\right)$ equations, respectively. Robust standard errors are given in parentheses. ^∗^ significant at 10*%* level; ^∗∗^ significant at 5*%* level.

Turning firstly to the results relating to participation, we find that, consistent with existing evidence, increasing age, being married, living in a capital city, and being a new migrant decrease the probability of participation. On the other hand, being male, having started drinking at a young age, having a tattoo or body piercing, and being of Aboriginal or Torres Strait Islander background are associated with higher participation (see, e.g., Cameron & Williams, 2001; Deschesnes, Finès, & Demers, 2006; Ramful & Zhao, 2009; Saffer & Chaloupka, 1999). In terms of education, we find those with higher qualifications are more likely to report participation. However, we do not find evidence of labour market status or income effect on participation. Consistent with the literature, cannabis use among peers and the decriminalisation laws also have a positive impact on participation (see, e.g., Cameron & Williams, 2001; Delaney, Harmon, & Wall, 2008; Farrelly, Bray, Zarkin, & Wendling, 2001; Kenkel, Reed III, & Wang, 2002; Pudney, 2004; Saffer & Chaloupka, 1999), although support for tighter drug laws is negatively related to participation.

Focusing on the misreporting equation, we find that age, being male, and living in a capital city are associated with a higher probability of truthful reporting whereas individuals from a single‐parent household and of aboriginal status are more likely to misreport. Interestingly, we find higher level of education to be also associated with a higher probability of misreporting. Although we might expect decriminalisation to increase honest reporting in light of reduced legal implications, it is nevertheless associated with higher probability of misreporting (presumably this variable is capturing other state/time effects not controlled for elsewhere).

In terms of the instruments in the misreporting equation, all four of them are statistically significant and negative. The results suggest that the presence of another person during the completion of the survey, or the provision of assistance during such, increases the probability of misreporting. Similarly, the CATI and face‐to‐face methods of interview (relative to drop‐and‐collect) also increases the probability of misreporting. Finally, if the individual demonstrated a lack of trust in the survey by, in general, refusing to give a response, he or she also had a higher probability of misreporting.

With respect to the levels of consumption, we find that being male, of aboriginal descent and being unemployed, all have statistically significant positive effects. Similarly, having started drinking at a young age, having a tattoo or body piercing, and peer drug use are also positively related to levels of drug consumption. According to the rational addiction model by Becker and Murphy (1988) , drug users are rational, forward looking utility maximizers who base consumption decisions on full knowledge of the consequences of addiction. Current consumption by a young adult raises the user's marginal utility of future use but also reduces the overall utility in the future, given that the rational user takes account of the addictive properties of drugs and their implications for future health and wealth. We thus allow for this nonlinear age‐consumption relationship through a quadratic specification for age. 6Ideally, this theory would be tested using panel data. Unfortunately, we have to proxy this within a cross‐sectional approach.


Our results indeed show evidence of an inverted U‐shaped distribution of levels of consumption with age. In other words, at both ends of the age distribution, individuals are associated with lower levels of consumption. Having young children in the household, being employed or a full‐time student, and having higher qualifications are all associated with lower levels of consumption. Although we do not find evidence of any significant impact of household income on participation or misreporting, we observe a general decline in the levels of use in the highest income groups.

Considering the price variables in the consumption equation (which act as identifying variables here), we find that the price effect of cannabis is positive and significant. It is important to note that the price of cannabis is strongly associated with quality (see, e.g., Cameron & Williams, 2001; Williams, 2004), and because we are unable to control for the price variation due to quality, a positive price effect could well be picking up the drug quality effect on participation. This counter‐intuitive price effect is, however, also found for several competing models (such as the generalised ordered probit [*G* *O* *P*] and correlated ZIOP [*Z* *I* *O* *P* *C*]; see Table 3 and the following section) and is therefore not an adverse finding of the current approach per se. The level of cannabis consumption is also responsive to heroin and speed prices suggesting that cannabis is an economic substitute to heroin but a complement to speed. However, the price effects of cocaine, tobacco, and alcohol are all statistically insignificant. In summary then, with at least two price variables exhibiting high levels of significance and along with similarly strong identifying instruments in the participation and misreporting equations, we are overall confident in our model results.

**Table 3:** Partial effects for selected models

	*G* *O* *P*			*Z* *I* *O* *P* *C*							*D* *Z* *I* *O* *P* *C*							
				Nonparticipation		Zero		Overall			Nonparticipation		Misreporting		Zero		Overall	
						consumption									consumption			
	Pr(y=0)			Pr(r=0)		$\mathrm{Pr}\left(r=1,\tilde{y}=0\right)$		Pr(y=0)			Pr(r=0)		Pr(r=1,m=0)		$\mathrm{Pr}(r=1,m=1,\tilde{y}=0)$		Pr(y=0)	
CONSTANT	0.375	(0.234)		0.105	(0.008)**	0.259	(0.179)	0.364	(0.179)**		0.129	(0.011)**	−0.026	(0.009)**	0.239	(0.147)	0.342	(0.147)**
STAGE	−0.061	(0.006)**		0.047	(0.003)**	−0.059	(0.005)**	−0.012	(0.006)*		0.062	(0.006)**	−0.009	(0.005)*	−0.037	(0.007)**	0.016	(0.009)*
STAGESQ	0.114	(0.006)**		‐	‐	0.068	(0.007)**	0.068	(0.007)**		‐	‐	‐	‐	0.043	(0.007)**	0.043	(0.007)**
MALE	−0.024	(0.002)**		−0.012	(0.003)**	−0.013	(0.002)**	−0.025	(0.002)**		−0.011	(0.003)**	−0.004	(0.002)**	−0.010	(0.002)**	−0.025	(0.002)**
MARRIED	0.024	(0.002)**		0.030	(0.003)**	0.001	(0.002)	0.031	(0.002)**		0.031	(0.004)**	0.000	(0.002)	0.001	(0.001)	0.032	(0.003)**
PRESCHOOL	0.011	(0.002)**		0.006	(0.003)*	0.007	(0.002)**	0.013	(0.003)**		0.008	(0.005)	0.001	(0.002)	0.005	(0.002)**	0.013	(0.003)**
SINGPAR	0.004	(0.002)*		0.002	(0.004)	0.003	(0.002)	0.005	(0.003)*		−0.007	(0.006)	0.003	(0.002)	0.002	(0.002)	−0.001	(0.004)
CAPITAL	−0.001	(0.001)		0.001	(0.002)	−0.002	(0.001)	−0.001	(0.002)		0.007	(0.004)**	−0.005	(0.002)**	−0.001	(0.001)	0.002	(0.002)
ATSI	−0.008	(0.005)*		0.006	(0.006)	−0.011	(0.005)**	−0.005	(0.006)		−0.030	(0.013)**	0.007	(0.006)	−0.006	(0.005)	−0.029	(0.013)**
WORK	0.002	(0.002)		−0.007	(0.003)**	0.006	(0.002)**	−0.001	(0.003)		−0.004	(0.005)	−0.002	(0.002)	0.005	(0.002)**	−0.001	(0.003)
STUDY	0.007	(0.003)**		−0.011	(0.007)	0.011	(0.003)**	0.000	(0.005)		0.006	(0.008)	−0.003	(0.003)	0.007	(0.002)**	0.010	(0.006)
UNEMP	−0.015	(0.004)**		−0.014	(0.005)**	−0.006	(0.004)	−0.019	(0.005)**		−0.012	(0.008)	−0.005	(0.004)	−0.004	(0.002)	−0.021	(0.007)**
DEGREE	0.002	(0.002)		−0.020	(0.004)**	0.016	(0.002)**	−0.004	(0.003)		−0.026	(0.005)**	0.006	(0.003)**	0.011	(0.002)**	−0.009	(0.003)**
DIPLOMA	−0.002	(0.002)		−0.008	(0.003)**	0.005	(0.002)**	−0.003	(0.002)		−0.014	(0.004)**	0.003	(0.002)	0.004	(0.001)**	−0.007	(0.003)**
YR12	−0.003	(0.002)		−0.009	(0.004)**	0.005	(0.002)**	−0.004	(0.003)		−0.011	(0.005)**	0.002	(0.002)	0.003	(0.002)**	−0.006	(0.004)*
HINC1	−0.009	(0.006)		0.005	(0.010)	−0.009	(0.005)*	−0.004	(0.008)		−0.004	(0.014)	0.005	(0.007)	−0.009	(0.005)*	−0.007	(0.011)
HINC2	−0.018	(0.005)**		−0.013	(0.007)*	−0.006	(0.005)	−0.019	(0.006)**		−0.012	(0.010)	−0.004	(0.005)	−0.005	(0.003)	−0.021	(0.009)**
HINC3	−0.015	(0.003)**		0.000	(0.005)	−0.012	(0.003)**	−0.012	(0.004)**		−0.003	(0.007)	−0.001	(0.003)	−0.009	(0.002)**	−0.013	(0.005)**
HINC4	−0.013	(0.003)**		0.000	(0.005)	−0.011	(0.003)**	−0.011	(0.004)**		−0.007	(0.007)	0.001	(0.003)	−0.008	(0.002)**	−0.014	(0.005)**
HINC5	−0.010	(0.002)**		0.006	(0.004)	−0.012	(0.002)**	−0.007	(0.003)**		0.001	(0.006)	−0.001	(0.002)	−0.009	(0.002)**	−0.008	(0.004)*
HINC6	−0.005	(0.002)**		0.005	(0.003)	−0.007	(0.002)**	−0.002	(0.003)		0.000	(0.005)	0.001	(0.002)	−0.006	(0.002)**	−0.004	(0.003)
HINC7	−0.003	(0.002)*		0.002	(0.003)	−0.004	(0.002)**	−0.001	(0.002)		0.003	(0.005)	−0.001	(0.002)	−0.003	(0.001)**	−0.001	(0.003)
DECRIM	−0.003	(0.002)		−0.004	(0.002)*	0.000	(0.002)	−0.005	(0.002)**		−0.013	(0.004)**	0.004	(0.002)**	−0.001	(0.001)	−0.009	(0.002)**
MIGR10	0.004	(0.003)		0.010	(0.005)*	−0.002	(0.003)	0.008	(0.004)*		0.018	(0.008)**	−0.005	(0.005)	−0.001	(0.002)	0.011	(0.005)**
YNGDRINK	−0.040	(0.002)**		−0.037	(0.004)**	−0.011	(0.003)**	−0.048	(0.002)**		−0.049	(0.003)**	‐	‐	−0.005	(0.002)**	−0.054	(0.003)**
YR04	−0.005	(0.002)**		−0.001	(0.003)	−0.002	(0.002)	−0.003	(0.003)		0.001	(0.003)	‐	‐	−0.001	(0.002)	0.000	(0.003)
YR07	0.000	(0.004)		0.002	(0.003)	0.002	(0.003)	0.004	(0.003)		0.008	(0.003)**	‐	‐	0.000	(0.002)	0.008	(0.003)**
TATTOO	−0.019	(0.002)**		−0.021	(0.003)**	−0.004	(0.002)**	−0.024	(0.002)**		−0.025	(0.003)**	‐	‐	−0.003	(0.001)**	−0.028	(0.003)**
PIERCING	−0.023	(0.002)**		−0.025	(0.004)**	−0.006	(0.002)**	−0.031	(0.003)**		−0.036	(0.004)**	‐	‐	−0.003	(0.002)**	−0.039	(0.004)**
PEER	−0.094	(0.004)**		−0.091	(0.005)**	−0.030	(0.003)**	−0.121	(0.005)**		−0.129	(0.009)**	‐	‐	−0.023	(0.004)**	−0.152	(0.008)**
CRIMSUP	0.084	(0.003)**		0.072	(0.006)**	0.027	(0.005)**	0.099	(0.003)**		0.087	(0.006)**	‐	‐	0.023	(0.005)**	0.109	(0.004)**
PRESENT	0.000	(0.000)		‐	‐	0.048	(0.006)**	0.048	(0.006)**		‐	‐	0.003	(0.001)**	‐	‐	0.003	(0.001)**
HELP	0.000	(0.000)		‐	‐	−0.007	(0.003)**	−0.007	(0.003)**		‐	‐	0.003	(0.002)**	‐	‐	0.003	(0.002)**
SURVTYPE	0.000	(0.000)		‐	‐	−0.001	(0.002)	−0.001	(0.002)		‐	‐	0.004	(0.002)**	‐	‐	0.004	(0.002)**
TRUST	0.000	(0.000)		‐	‐	−0.005	(0.002)**	−0.005	(0.002)**		‐	‐	0.031	(0.012)**	‐	‐	0.031	(0.012)**
LRPMAR	−0.010	(0.005)*		‐	‐	−0.010	(0.004)**	−0.010	(0.004)**		‐	‐	‐	‐	−0.010	(0.003)**	−0.010	(0.003)**
LRPCOC	−0.003	(0.004)		‐	‐	−0.001	(0.003)	−0.001	(0.003)		‐	‐	‐	‐	0.001	(0.002)	0.001	(0.002)
LRPHER	−0.010	(0.004)**		‐	‐	−0.007	(0.003)**	−0.007	(0.003)**		‐	‐	‐	‐	−0.005	(0.002)**	−0.005	(0.002)**
LRPSPD	0.004	(0.002)*		‐	‐	0.003	(0.002)	0.003	(0.002)		‐	‐	‐	‐	0.002	(0.001)	0.002	(0.001)
LRPTOB	0.004	(0.025)		‐	‐	−0.015	(0.019)	−0.015	(0.019)		‐	‐	‐	‐	−0.017	(0.015)	−0.017	(0.015)
LRPALC	−0.032	(0.023)		‐	‐	−0.019	(0.017)	−0.019	(0.017)		‐	‐	‐	‐	−0.017	(0.014)	−0.017	(0.014)

*Note*. Robust standard errors are given in parentheses. ^∗^ significant at 10 *%* level; ^∗∗^ significant at 5*%* level.

### 3.3 Partial effects

As with any probability model, partial effects are generally more informative than coefficients. There are several sets of partial effects that may be estimated here. For example, one may be interested in the partial effects of an explanatory variable on probabilities such as the probability of participation, *P* *r*(*r*=1), the probability of misreporting, *P* *r*(*m*=0), the probabilities for the levels of consumption *conditional* on participation, $Pr(\tilde{y}=j|r=1)$, and the overall probabilities for different levels of consumption, *P* *r*(*y*=*j*).

In particular, we are interested in the probability of reporting zeros, as this forms the major contribution of our approach. The partial effect on the overall probability of observing zero consumption, *P* *r*(*y*=0), is the sum of the effects on the probabilities of the three types of zeros; that is, the probability of nonparticipation, the probability of misreporting, and the probability of zero‐consumption arising from participants who are infrequent or potential consumers. Note that the explanatory variables of interest may appear in only one or two of **x**_*r*_, **x**_*m*_, and **x**_*y*_, or in all three. For comparison purposes, in Table 3, we also present results from a *G* *O* *P* model without any hurdles, where the boundary parameters are specified as a function of variables in **x**_*m*_ that do not appear in **x**_*y*_. Standard errors of the partial effects for all models are obtained using the *Delta* method (Greene, 2008), and the effects themselves were computed numerically.

We report the partial effects on *P* *r*(*y*=0)(estimated at sample means) coming from these three sources in the correlated *D* *Z* *I* *O* *P* *C* model in Table 3. For a further comparison, we also compare these results with partial effects estimated from a *Z* *I* *O* *P* *C* model that allows zero observations to come from two distinct sources: nonparticipation and infrequent consumption/misreporting; and, as noted, from a *G* *O* *P* model that does not explicitly model zero observations coming from different sources but allows for the boundary parameters inherent in the *O* *P* model to be a function of the *zero‐generating* variables. 7Specifically, we allow the boundary or threshold parameters of the OP model, which are generally constants in a standard OP, to be a function of instruments that we used in the misreporting equation.For the *D* *Z* *I* *O* *P* *C* model, the overall partial effects are decomposed in three parts: nonparticipation, *P* *r*(*r*=0), with clearly *participation* being the mirror image of this; participation and misreporting, *P* *r*(*r*=1,*m*=0); and participation, truthful reporting, and zero consumption, $Pr(r=1,m=1,\tilde{y}=0)$. In contrast, we have two components in the *Z* *I* *O* *P* *C* model: nonparticipation, *P* *r*(*r*=0); and participation and zero consumption, $Pr(r=1,\tilde{y}=0)$.

Interestingly, we observe some important differences across the estimates from the alternate models for some explanatory variables such as living in a capital city, household income, and education. A key example is the effect of education. The *Z* *I* *O* *P* *C* model indicates that those with higher qualifications have a lower probability of nonparticipation but a higher probability of participation with infrequent consumption. With an additional misreporting dimension in the *D* *Z* *I* *O* *P* *C* model, we find that those with higher qualifications also have a higher probability of misreporting. For instance, from the *Z* *I* *O* *P* *C* results, relative to those with less than year 12 qualifications, degree holders have a 2.0 percentage point (pp) lower probability of being a nonparticipant and a 1.6 pp higher probability of being a participant with zero consumption, resulting in an overall 0.4 pp lower probability of observing zero consumption. The *D* *Z* *I* *O* *P* *C* results, in contrast, indicate that degree holders have a 2.6 pp lower probability of being a nonparticipant, a 1.1 pp higher probability of being a participant with zero consumption and also a 0.6 pp higher probability of being a misreporter. Overall, degree holders have a 0.9 pp net negative effect on the probability of observing zero consumption relative to those with less than year 12 qualifications. Finally, basing policy advice on the *G* *O* *P* model results, one would conclude that education has no impact on cannabis consumption, presumably with the opposing effects being cancelled out.

The effect of decriminalisation also highlights the potentials of the *D* *Z* *I* *O* *P* *C* model. For instance, the *G* *O* *P* indicates that decriminalisation does not affect consumption. From the *Z* *I* *O* *P* *C* model, we find that decriminalisation is associated with higher probabilities of participation, whereas the *D* *Z* *I* *O* *P* *C* suggests that an easing of the criminal justice system is actually associated with both a higher probability of participation and a higher probability of misreporting.

In short, as a result of comparisons of models that ignore any potential misreporting effects, it appears that such models result in biased estimates of various quantities of interest, and potentially erroneous policy advice.

### 3.4 Robustness checks

There may be concerns that certain control variables may be endogenous and/or that some of the instruments we use to identify the model in various equations may have effects elsewhere in the model. 8Although we note that the important variables that identify the misreporting equation are all survey‐related, which make them unlikely to be related to drug consumption, providing a strong ground for identification, the importance of these factors in the misclassification literature and their statistical significance in the estimated model lend further support to their inclusion in the misreporting equation. However, testing the validity of instruments in nonlinear models is a difficult task (see, e.g., Davidson & MacKinnon, 1993).In this section, we perform a robustness check to test whether our results change significantly with the exclusion of the so‐called problem variables and alternate specifications. We look at four different specifications that we compare with our main one: Spec 1 – we enter price variables in both the participation and the consumption equations; Spec 2 – we run the main specification without *tattoo* and *body piercing*; Spec 3 – we run the main specification without *peer influence*; and Spec 4 – we run the main specification with only *survey type* and *trust*as identifying variables in the misreporting equation.

Comparing across the resulting partial effects, we find that the results are generally robust to the various specifications (Table 4). For example, the partial effect of male is −0.025 from the main model. This effect varies between −0.023 and −0.030 across the four alternative specifications. Similarly, the partial effect of having a degree varies in the range −0.004 to −0.009, comparable with the estimated partial effect of −0.009 from the main model. With regard to the results for the respective misreporting equations, the results essentially remained unchanged (and are available on request).

**Table 4:** Comparison across specifications

	Main Spec			Spec 1			Spec 2			Spec 3			Spec 4	
	Pr(r=0)			Pr(r=0)			Pr(r=0)			Pr(r=0)			Pr(r=0)	
CONSTANT	0.342	(0.147)**		0.292	(0.342)		0.451	(0.207)**		0.511	(0.207)**		0.403	(0.185)**
STAGE	0.016	(0.009)*		0.017	(0.010)*		0.011	(0.012)		0.006	(0.012)		−0.005	(0.009)
STAGESQ	0.043	(0.007)**		0.042	(0.008)**		0.065	(0.009)**		0.064	(0.005)**		0.065	(0.009)**
MALE	−0.025	(0.002)**		−0.024	(0.002)**		−0.025	(0.003)**		−0.030	(0.003)**		−0.023	(0.002)**
MARRIED	0.032	(0.003)**		0.032	(0.003)**		0.036	(0.003)**		0.040	(0.003)**		0.034	(0.003)**
PRESCHOOL	0.013	(0.003)**		0.013	(0.003)**		0.017	(0.003)**		0.017	(0.003)**		0.015	(0.003)**
SINGPAR	−0.001	(0.004)		−0.002	(0.004)		0.000	(0.004)		−0.002	(0.004)		−0.001	(0.004)
CAPITAL	0.002	(0.002)		0.002	(0.002)		0.001	(0.002)		0.000	(0.002)		0.001	(0.002)
ATSI	−0.029	(0.013)**		−0.028	(0.011)**		−0.035	(0.013)**		−0.030	(0.013)**		−0.034	(0.011)**
WORK	−0.001	(0.003)		−0.001	(0.003)		0.002	(0.003)		0.003	(0.003)		0.000	(0.003)
STUDY	0.010	(0.006)		0.010	(0.006)*		−0.012	(0.011)		−0.001	(0.011)		0.004	(0.008)
UNEMP	−0.021	(0.007)**		−0.021	(0.006)**		−0.017	(0.006)**		−0.025	(0.006)**		−0.019	(0.006)**
DEGREE	−0.009	(0.003)**		−0.009	(0.003)**		−0.005	(0.003)		−0.004	(0.003)		−0.008	(0.003)**
DIPLOMA	−0.007	(0.003)**		−0.006	(0.004)*		−0.004	(0.004)		−0.003	(0.004)		−0.007	(0.003)*
YR12	−0.006	(0.004)*		−0.007	(0.003)**		−0.006	(0.003)**		−0.007	(0.003)**		−0.007	(0.003)**
HINC1	−0.007	(0.011)		−0.007	(0.011)		−0.008	(0.010)		−0.016	(0.010)		−0.003	(0.009)
HINC2	−0.021	(0.009)**		−0.021	(0.007)**		−0.023	(0.007)**		−0.025	(0.007)**		−0.019	(0.007)**
HINC3	−0.013	(0.005)**		−0.013	(0.005)**		−0.017	(0.005)**		−0.020	(0.005)**		−0.011	(0.005)**
HINC4	−0.014	(0.005)**		−0.014	(0.005)**		−0.013	(0.005)**		−0.019	(0.005)**		−0.013	(0.005)**
HINC5	−0.008	(0.004)*		−0.008	(0.003)**		−0.009	(0.004)**		−0.012	(0.004)**		−0.008	(0.004)**
HINC6	−0.004	(0.003)		−0.004	(0.003)		−0.006	(0.003)*		0.000	(0.003)		−0.003	(0.003)
HINC7	−0.001	(0.003)		−0.001	(0.003)		−0.002	(0.003)		−0.001	(0.003)		−0.002	(0.003)
DECRIM	−0.009	(0.002)**		−0.005	(0.003)*		−0.010	(0.003)**		−0.009	(0.003)**		−0.008	(0.003)**
MIGR10	0.011	(0.005)**		0.012	(0.005)**		0.006	(0.006)		0.008	(0.006)		0.010	(0.005)**
YNGDRINK	−0.054	(0.003)**		−0.054	(0.003)**		−0.052	(0.003)**		−0.067	(0.003)**		−0.052	(0.003)**
YR04	0.000	(0.003)		−0.006	(0.004)*		−0.002	(0.003)		−0.001	(0.003)		−0.002	(0.003)
YR07	0.008	(0.003)**		0.000	(0.006)		0.003	(0.004)		0.007	(0.004)*		0.005	(0.004)
TATTOO	−0.028	(0.003)**		−0.028	(0.003)**		‐	‐		−0.042	(0.013)**		−0.027	(0.003)**
PIERCING	−0.039	(0.004)**		−0.040	(0.004)**		‐	‐		−0.055	(0.005)**		−0.039	(0.004)**
PEER	−0.152	(0.008)**		−0.151	(0.008)**		−0.168	(0.013)**		‐	‐		−0.151	(0.010)**
CRIMSUP	0.109	(0.004)**		0.109	(0.004)**		0.120	(0.005)**		0.124	(0.001)**		0.107	(0.004)**
PRESENT	0.003	(0.001)**		0.003	(0.001)**		0.001	(0.001)		0.006	(0.001)**		‐	‐
HELP	0.003	(0.002)**		0.003	(0.002)**		0.002	(0.001)**		0.000	(0.002)		‐	‐
SURVTYPE	0.004	(0.002)**		0.004	(0.002)*		0.003	(0.002)**		0.010	(0.006)*		0.004	(0.001)**
TRUST	0.031	(0.012)**		0.030	(0.012)**		0.019	(0.006)**		0.058	(0.009)**		0.022	(0.007)**
LRPMAR	−0.010	(0.003)**		−0.007	(0.008)		−0.011	(0.005)**		−0.019	(0.003)**		−0.011	(0.004)**
LRPCOC	0.001	(0.002)		−0.003	(0.005)		−0.002	(0.003)		0.000	(0.003)		0.000	(0.003)
LRPHER	−0.005	(0.002)**		−0.016	(0.006)**		−0.008	(0.003)**		−0.017	(0.002)**		−0.007	(0.003)**
LRPSPD	0.002	(0.001)		0.005	(0.003)		0.002	(0.002)		0.007	(0.022)		0.003	(0.002)
LRPTOB	−0.017	(0.015)		0.029	(0.036)		−0.026	(0.022)		−0.022	(0.020)		−0.018	(0.019)
LRPALC	−0.017	(0.014)		−0.051	(0.034)		−0.024	(0.020)		−0.035	(0.020)*		−0.024	(0.018)

*Note*. Robust standard errors are given in parentheses. ^∗^ significant at 10 *%* level; ^∗∗^ significant at 5*%* level. The four specifications are similar to the main one except for the following: Spec 1 – price variables appear in both participation and consumption equations; Spec 2 – tattoo and body piercing are dropped from the main specification; Spec 3 – peer influence is dropped from the main specification; and Spec 4 – only survey type and trust are used as instruments in the misreporting equation.

Different specifications for the misreporting equation were also experimented with; for example, it could be argued that the *peer influence* and *support for criminalisation* variables also affect the misreporting process. These results are not presented here (and are available on request), but again essentially did not significantly affect the overall results. Although we argue above that a positive effect of own‐price on consumption levels might be picking‐up quality effects, it could also be argued that there is a potential for reverse causation here. The same arguments could be made about the findings with regard to decriminalisation. In light of this, we also experimented with replacing these variables with both year and state dummies. Again, although not presented here, once more, the general results were not unduly affected.

We note here that there is also information available in the data regarding lifetime cannabis use and also use in the last month. One would expect misreporting levels to be less for the former and stronger for the latter (Brown, Harris, Srivastava, & Zhang, 2017). However, unfortunately, these are simple zero/one responses, so that they do not fit into the framework proposed in this paper.

Finally, although we do report the results for reasons of space, we also conducted a series of Monte Carlo experiments based on the empirical data and specification used in the paper. In short, the model performed extremely well (with regard to estimating all key quantities of interest) under a range of scenarios and did not suggest any identification issues. A strong finding was that it appeared most important to ensure identifying variables in the misreporting equation. Another very strong finding, on the other hand, was that the restricted submodels (*O* *P*,*G* *O* *P*,*Z* *I* *O* *P*) yielded heavily biased estimates of some key quantities of interest.

### 3.5 Predicted probabilities

A key output from such a model relates to summary predicted probabilities especially with regard to the zeros. Thus, there are several predicted probabilities that will be of interest with the *D* *Z* *I* *O* *P* class of models. For example, one may be interested in the partial probability of participation, *P* *r*(*r*=1). In terms of misreporting, one may be interested in the partial probability of misreporting, *P* *r*(*m*=0); or the joint probability of participation and misreporting, *P* *r*(*r*=1,*m*=0); or the probability of truthful reporting, conditional on participation, *P* *r*(*m*=1|*r*=1). Similarly, there is a range of probabilities one may be interested in predicting levels of consumption. However, our main interest in this paper is on the misreporting dimension. Therefore, to gain insight into the sources of the observed zeros, we present in the first row in Table 5 the predicted probability (averaged across all individuals) of the zeros and its three respective components (using the equations presented above): nonparticipation, misreporting, and zero consumption. We find that the overall predicted probability of 88.8% of observed zero consumption in the population is made up of the respective probability of 81.5% nonparticipation, 4.4% misreporting, and 2.9% infrequent consumption.

**Table 5:** Predicted zero probabilities

	Non‐participation	Misreporting	Zero consumption	Full
Marginal probability	0.815	0.044	0.029	0.888
of zero consumption	(0.011)**	(0.008)**	(0.007)**	(0.001)**
Posterior probability	0.722	0.165	0.113	1
of zero consumption	(0.028)**	(0.022)**	(0.026)**	

*Note*. Robust standard errors are given in parentheses.^∗∗^ significant at 5*%* level.

Such probabilities can be thought of as prior probabilities. That is, they apply to a randomly selected individual from the population, about whom we know nothing except for their characteristics. However, to provide further insights into the extent of misreporting, it is possible to estimate posterior probabilities, analogous to those considered in latent class models (Greene, 2008) that are conditional on the outcome chosen by the individual. This specifically allows us to make a prediction on what percentage of these zeros come from nonparticipation, misreporting, and zero consumption, respectively, using all the information we have on the individual. It therefore attempts to answer the following question: *given that an individual recorded a zero, what is the probability that he/she is a true non‐participant or a misreporting participant or an infrequent consumer (given their observed characteristics)?* The posterior probabilities for the three types of zeros are given as (Greene, 2008):

(13) $$\begin{array}{ccc} Pr(r & =0|x,y=0)=\frac{f(r=0|x)}{f(y=0|x)} & \\ & =\frac{1-\Phi \left(x_{r}^{\prime }\beta_{r}\right)}{\left[1-\Phi \left(x_{r}^{\prime }\beta_{r}\right)\right]+\Phi_{2}\left(x_{r}^{\prime }\beta_{r},-x_{m}^{\prime }\beta_{m};\Omega_{2}\right)+\Phi_{3}\left(x_{r}^{\prime }\beta_{r},x_{m}^{\prime }\beta_{m},-x_{y}^{\prime }\beta_{y};\Omega_{3}\right)} \end{array}$$

(14) $$\begin{array}{ccc} \;Pr(r & =1,m=0|x,y=0)=\frac{f(r=1,m=0|x)}{f(y=0|x)} & \\ & \;=\frac{\Phi_{2}\left(x_{r}^{\prime }\beta_{r},-x_{m}^{\prime }\beta_{m};\Omega_{2}\right)}{\left[1-\Phi \left(x_{r}^{\prime }\beta_{r}\right)\right]+\Phi_{2}\left(x_{r}^{\prime }\beta_{r},-x_{m}^{\prime }\beta_{m};\Omega_{2}\right)+\Phi_{3}\left(x_{r}^{\prime }\beta_{r},x_{m}^{\prime }\beta_{m},-x_{m}^{\prime }\beta_{y};\Omega_{3}\right)} \end{array}$$

(15) $$\begin{array}{ccc} \;Pr(r & =1,m=1,\tilde{y}=0|x,y=0)=\frac{f(r=1,m=1,\tilde{y}=0|x)}{f(y=0|x)} & \\ & \;=\frac{\Phi_{3}\left(x_{r}^{\prime }\beta_{r},x_{m}^{\prime }\beta_{m},-x_{m}^{\prime }\beta_{y};\Omega_{3}\right)}{\left[1-\Phi \left(x_{r}^{\prime }\beta_{r}\right)\right]+\Phi_{2}\left(x_{r}^{\prime }\beta_{r}1-x_{m}^{\prime }\beta_{m};\Omega_{2}\right)+\Phi_{3}\left(x_{r}^{\prime }\beta_{r},x_{m}^{\prime }\beta_{m},-x_{m}^{\prime }\beta_{y};\Omega_{3}\right)} \end{array}$$

Note that we have used the uncorrelated DZIOP model in the above. Note also the above probabilities are all conditional on *y*=0. As a result, the numerator on the right‐hand side of all three equations should be joint probabilities with *y*=0. For example, the numerator in Equation 13 is strictly *f*(*r*=0,*y*=0|**x**). However, this can be simplified as above given *y*=0 when *r*=0. The same applies in the other two equations given *y*=0 when *m*=0 or when $\tilde{y}=0$.

From Table 5, we find that about 72% of the reported zeros come from genuine nonparticipation, 17% from those who have misreported their participation, and 11% from zero consumption participants (estimated individually and averaged across). Moreover, the small estimated standard errors on these quantities is an indication that they have been estimated relatively accurately. These are important findings suggesting that misreporting and infrequent users reporting zero consumption in survey data with a fixed time frame (12 months here) may lead to considerable underestimation of drug use prevalence.

### 3.6 Conclusions

When modelling “social bads”, such as illegal drug consumption, researchers are often faced with a dependent variable characterised by a large number of zero observations. Such zero observations could result from individuals misreporting activities regarded as being socially undesirable, illegal, or which are associated with perceived social stigma, as is the case with drug‐consumption. The accuracy of the information gathered from surveys is therefore crucially dependent on the respondents providing reliable and accurate responses. If not, important behaviours will be misclassified thereby masking the true incidence of such. Thus, if ignored, misreporting potentially leads to inaccurate estimates of the prevalence of such behaviours and ultimately may lead one to question the validity of any conclusions drawn on the basis of these, which in turn raises concerns as to how useful such data actually is to policy‐makers.

Building on the recent literature on hurdle and double‐hurdle models, we propose a double‐inflated modelling framework, where the zero observations are allowed to come from the following: nonparticipants; participant misreporters (who have larger loss functions associated with a truthful response); and infrequent consumers. Due to our empirical application, the model is derived for the case of an ordered discrete dependent variable. However, it is similarly possible to augment other such zero‐inflated models (e.g., zero‐inflated count models, and double‐hurdle models for continuous variables). The model is then applied to a consumer choice problem of cannabis consumption.

Overall, the results suggest that misreporting has a significant effect on the incidence of cannabis use. Specifically, the posterior probability of misreporting cannabis participation is estimated at 0.17. In other words, out of all the reported zeros, 17% can be accounted to misreporting. The model also predicts that 11% of the reported zeros for cannabis use are from infrequent users with zero consumption (“corner solution” individuals), and only 72% are from true nonparticipants. The modelling framework also provides important insights into the drivers of misreporting in surveys compared to more standard modelling techniques. For example, a zero effect in a simple model might well disguise significant composite effects that simply net each other out. Interestingly, the findings also suggest that the extent of misreporting is influenced by how the survey was administered as well as factors such as the presence of other individuals when the survey was completed, and the individual's general trust in such surveys. In order to enhance accuracy of information gathered from surveys, it is therefore important to pay attention to the conditions under which the survey data are collected. The findings suggest that accounting for misreporting is important in the context of using survey data related to sensitive activities, especially where such data are used to inform public policy.

## APPENDIX 1 DEFINITION OF VARIABLES

### 1.1

- *y*: Levels of cannabis/cannabis consumption; *y*=0 if not current user, *y*=1 if using cannabis/cannabis once or twice a year, *y*=2 if using cannabis/cannabis monthly or every few months, and *y*=3 if using cannabis/cannabis everyday or once a week.
- **STAGE**: standardised age.
- **STAGESQ**: standardised age‐squared .
- **MALE**: =1 for male; and =0 for female.
- **MARRIED**: =1 if married or de facto; and =0 otherwise.
- **PRESCHOOL**: =1 if the respondent has pre‐school aged child/children, and =0 otherwise.
- **SINGPAR**: 1 if respondent comes from a single parent household, and =0 otherwise.
- **CAPITAL**: =1 if the respondent resides in a capital city, and =0 otherwise.
- **ATSI**: =1 if respondent is of Aboriginal or Torres Strait Islander origin, and =0 otherwise.
- **WORK**: =1 if mainly employed; and =0 otherwise.
- **UNEMP**=1 if unemployed; and =0 otherwise.
- **STUDY**: =1 if mainly study; and =0 otherwise.
- **OTHER**=1 if retired, home duty, or volunteer work; and =0 otherwise. This variable is used as the base of comparison for work status dummies and is dropped in the estimation.
- **DEGREE**: =1 if the highest qualification is a tertiary degree, and =0 otherwise.
- **DIPLOMA**:=1 if the highest qualification is a non‐tertiary diploma or trade certificate, and =0 otherwise.
- **YR12**: =1 if the highest qualification is Year 12, and =0 otherwise.
- **LESSYR12**: =1 if the highest qualification is below Year 12, and =0 otherwise. This variable is used as the base of comparison for education dummies and is dropped in the estimation.
- **HINC**: Household income before tax, measured in thousands of Australian dollars where **HINC1**=1 for $0‐$9,999; **HINC2**=1 for $10,000‐$19,999; **HINC3**=1 for $20,000‐$29,999; **HINC4**=1 for $30,000‐$39,999; **HINC5**=1 for $40,000‐$59,999; ** HINC6**=1 for $60,000‐$89,999; **HINC7**=1 for $90,000‐$99,999; **HINC8**=1 for $100,000 and above. **HINC8** is used as the base of comparison for income and is dropped in the estimation.
- **DECRIM**: =1 if respondent resides in a state where small possession is decriminalised and =0 otherwise.
- **MIGR10**: =1 if migrated to Australia in the last 10 years, and =0 otherwise.
- **YNGDRINK**: = 1 if started drinking at the age of 12, and = 0 otherwise.
- **TATTOO**: =1 if undergone any tattoo procedure, and =0 otherwise.
- **BODYPIER**: =1 if undergone any body piercing procedure, and =0 otherwise.
- **PEER**: =1 if most or all of respondent's friends and acquaintances use cannabis/cannabis.
- **LRPMAR**: Logarithm of real price for cannabis measured in dollars per ounce.
- **LRPCOC**: Logarithm of real price of cocaine measured in dollars per gram.
- **LRPSPD**: Logarithm of real price of speed measured in dollars per gram.
- **LRPHER**: Logarithm of real price of heroin measured in dollars per gram.
- **LRPTOB**: Logarithm of real price index for tobacco.
- **LRPALC**: Logarithm of real price index for alcoholic drinks.
- **YGPMAR**: Cross product of cannabis price with an indicator that the respondent is under 18.
- **YGPTOB**: Cross product of tobacco price with an indicator that the respondent is under 18.
- **CRIMSUP**: =1 if respondent believes small quantity of cannabis for personal use should be a criminal offence; and =0 otherwise.
- **PRESENT**: =1 if anyone else was present when the respondent was completing the survey questionnaire; and =0 otherwise.
- **HELP**: =1 if anyone helped the respondent complete the survey questionnaire; and =0 otherwise.
- **SURVTYPE**: =1 if the computer‐assisted telephone interview (CATI) method or face‐to‐face method was used to collect data; and =0 if drop and collect method was used.
- **TRUST**: percentage of compulsory questions left unanswered in the survey.
